# 3D-Printable Hierarchical Nanogel-GelMA Composite Hydrogel System

**DOI:** 10.3390/polym13152508

**Published:** 2021-07-29

**Authors:** Guangyue Zu, Marnix Meijer, Olga Mergel, Heng Zhang, Patrick van Rijn

**Affiliations:** 1 University of Groningen, University Medical Center Groningen, Department of Biomedical Engineering, W. J. Kolff Institute for Biomedical Engineering and Materials Science, A. Deusinglaan 1, 9713 AV Groningen, The Netherlands; g.zu@umcg.nl (G.Z.); marnix-meijer@hotmail.com (M.M.); Olga.Mergel@rwth-aachen.de (O.M.); 2 University of Groningen, Zernike Institute for Advanced Materials, Nijenborgh 4, 9747 AG Groningen, The Netherlands; h.zhang@rug.nl

**Keywords:** nanogels, GelMA hydrogels, 3D printing, hierarchical network, ECM

## Abstract

The strength of the extracellular matrix (ECM) is that it is hierarchical in terms of matrix built-up, matrix density and fiber structure, which allows for hormones, cytokines, and other small biomolecules to be stored within its network. The ECM-like hydrogels that are currently used do not possess this ability, and long-term storage, along with the need for free diffusion of small molecules, are generally incompatible requirements. Nanogels are able to fulfill the additional requirements upon successful integration. Herein, a stable hierarchical nanogel–gelatin methacryloyl (GelMA) composite hydrogel system is provided by covalently embedding nanogels inside the micropore network of GelMA hydrogel to allow a controlled local functionality that is not found in a homogenous GelMA hydrogel. Nanogels have emerged as a powerful tool in nanomedicine and are highly versatile, due to their simplicity of chemical control and biological compatibility. In this study, an *N*-isopropylacrylamide-based nanogel with primary amine groups on the surface was modified with methacryloyl groups to obtain a photo-cross-linking ability similar to GelMA. The nanogel-GelMA composite hydrogel was formed by mixing the GelMA and the photo-initiator within the nanogel solution through UV irradiation. The morphology of the composite hydrogel was observed by scanning electron microscopy, which clearly showed the nanogel wrapped within the GelMA network and covering the surface of the pore wall. A release experiment was conducted to prove covalent bonding and the stability of the nanogel inside the GelMA hydrogel. In addition, 3D printability studies showed that the nanogel-GelMA composite ink is printable. Therefore, the suggested stable hierarchical nanogel-GelMA composite hydrogel system has great potential to achieve the in situ delivery and controllable release of bioactive molecules in 3D cell culture systems.

## 1. Introduction

Three-dimensional (3D) cell culture has shown great potential in the fields of tissue engineering, wound healing, and drug screening because its structure is more compatible with human physiology, and because of its ability to be designed to mimic the characteristics of the native extracellular matrix (ECM) [1,2]. Besides serving as a scaffold, the ECM also acts as a biological and mechanical support that regulates the ability of the cells to survive, migrate, differentiate, and form a desired 3D tissue architecture [2,3]. The 3D concept has been adopted in bioprinting approaches, where the cell culture scaffold is created in a layer-by-layer fashion with pre-seeded cells within the matrix. Bioprinting has been widely accepted, due to the accurate fabrication of complex constructs of cells and hydrogels to achieve spatially controlled 3D constructs that mimic the function of certain tissues, leading to the direct manufacturing of mature artificial tissue in vitro that is suitable for transplantation [4,5,6,7].

One of the most appealing biomaterials that is currently being applied in 3D cell culture is gelatin methacryloyl-based hydrogel, which is usually abbreviated as GelMA hydrogel [8,9,10]. One can easily prepare GelMA by modifying the lysine residues in gelatin with methacrylic anhydride (MA) to confer on the gelatin the property of photo-cross-linking, by the introduction of methacryloyl substituent groups. The functional amino acid motifs of gelatin, such as the arginine–glycine–aspartic acid (RGD) sequences that promote cell attachment, will not be influenced in this process [11,12]. Conveniently, GelMA hydrogel can be obtained by photo-polymerization of the methacryloyl substituents, irradiating by blue or UV light with the assistance of a proper photo-initiator [7,13,14]. In addition, GelMA hydrogel exhibits excellent biocompatibility, degradability, and processibility, which makes it an excellent candidate in tissue engineering [15,16]. For example, Lin et al. successfully injected a liquid solution of GelMA containing human blood-derived endothelial colony-forming cells and bone marrow-derived mesenchymal stem cells into the subcutaneous space of an immuno-deficient mouse, then rapidly cross-linked by transdermal exposure to UV light, forming a 3D cell-laden polymerized construct. The implanted human cells subsequently generated an extensive vasculature and were uniformly distributed throughout the construct [17]. However, it is not only the encapsulation of stem cells in a 3D culture system that is essential for therapeutic applications like bone tissue engineering; the in situ delivery of localized, sustained bioactive molecules, such as nutrients, drugs, and growth factors, also plays an important role [18,19]. However, the highly hydrated, porous structure of GelMA hydrogels with micrometer range aqueous pore size results in the rapid diffusive loss of entrapped biomolecules under physiological conditions, which limits their uses in long-term biomedical applications [20,21].

Hierarchical structures are considered to be a good solution to solve the problem, namely, to introduce nano-sized carriers into the micro-sized porous structure of GelMA hydrogel. For example, Elkhoury et al. recently reported a method of embedding bioactive naringin-loaded salmon-derived lecithin nanosized liposomal building blocks inside GelMA hydrogels [22]. The controlled release was successfully realized, which has proven the efficiency of a hierarchical structure. However, the micro–nano interactions in most cases are non-covalent, which means that the nanocarriers are not stable and may escape from the hydrogel matrix. To avoid this downside, a GelMA-compatible one-step approach for introducing a covalently bound hydrogel–nanocarrier system would offer a potential solution.

Herein, we propose a 3D-printable hierarchical system, with nanogels covalently embedding inside the GelMA network without affecting the overall printing process, that allows for the introduction of a hierarchical build-up of sophisticated functions, such as storage, imaging, and delivery. A nanogel is a cross-linked polymer network, with the size being tunable between tens of nanometers to several hundred nanometers, and can be swollen by solvent, structuring a dense core but a fuzzy surface [23]. Notably, they may be designed to be responsive to several stimuli, such as temperature, pH, ionic strength, redox chemistry, and UV light [24,25,26,27], which makes them a perfect toolbox for small bioactive molecule encapsulation and controlled release and interface alterations [23,28,29,30,31]. The *N*-isopropylacrylamide (NIPAM)-based nanogel was used in this work. Primary amine groups were introduced by the copolymerization of *N*-(3-aminopropyl)methacrylamide hydrochloride (APMA) comonomers on the surface of the nanogels, which were further modified with methacryloyl groups to confer the photo-cross-linking property to the nanogel that is compatible with GelMA cross-linking. By simply mixing GelMA, the nanogel and the photo-initiator at a certain ratio, the solution was ready to form the composite hydrogel by irradiating with UV light. A series of measurements were performed to study the behavior of the system, and the results support the covalent bonding strategy and the stability of the nanogels within the GelMA hydrogel. This strategy will open up future research on building stable, hierarchically structured hydrogel systems for biomedical applications such as therapeutic tissue engineering, where the nanogel toolbox can be deployed to enhance the function of GelMA-based bioprinting and tissue engineering approaches.

## 2. Materials and Methods

### 2.1. Materials

Gelatin (porcine type A with 250 bloom) was purchased from Gelita, Eberbach, Germany. Methacrylic anhydride (MA, 94%), 2,2′-Azobis(2-methylpropionamidine) dihydrochloride (AMPA V50, 97%), *N*,*N*′-methylene-bis(acrylamide) (BIS, 99%), hexadecyltrimethylammonium bromide (CTAB, 99%), and deuterium oxide (D_2_O, 99.9%) were purchased from Sigma-Aldrich, Zwijndrecht, The Netherlands. *N*-isopropylacrylamide (NIPAM, 98%) was purchased from TCI, Zwijndrecht, Belgium. *N*-(3-aminopropyl)methacrylamide hydrochloride (APMA, 98%), and methacryloxyethyl thiocarbamoyl rhodamine B (MRB, 95%) were purchased from Polysciences, Bergstrasse, Germany. Lithium phenyl-2,4,6-trimethylbenzoylphosphinate (LAP, 95%) was purchased from Allevi, Inc., PA, USA. Hydrogen chloride (HCl), sodium bicarbonate (NaHCO_3_), and sodium hydroxide (NaOH) were purchased from Merck, Darmstadt, Germany. All chemicals were used as received, without any further purification. Ultrapure water (18.2 MΩ, Arium 611 DI water purification system; Sartorius AG, Göttingen, Germany) was used in all experiments.

### 2.2. Synthesis of Gelatin Methacryloyl (GelMA)

**Figure 1:**
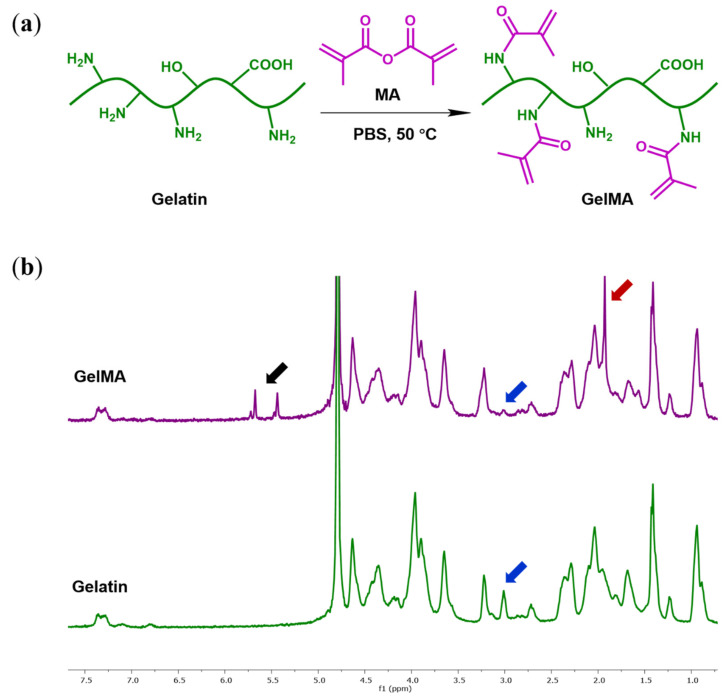
(**a**) Synthesis route of GelMA; (**b**) ^1^H-NMR spectra of GelMA and gelatin.

GelMA was synthesized as described previously [32], as shown in Figure 1a. Briefly, 10 g gelatin was dissolved in 100 mL phosphate-buffered saline (PBS, pH = 7.4) at 10% (*w/v*) and heated to 50 °C, and 6 g MA was added slowly dropwise and reacted for 6 h under constant stirring. The reaction was stopped by adding 200 mL PBS. The mixture was then transferred to dialysis tubing (MWCO = 3500 Da, Sigma-Aldrich, Zwijndrecht, The Netherlands) and dialyzed against ultrapure water at 35 °C for 7 days, the dialysis water was changed twice daily. The purified GelMA solution was freeze-dried and stored at −80 °C for further use.

### 2.3. ^1^H-NMR of Gelatin and GelMA

To determine the degree of functionalization of GelMA, the ^1^H-NMR spectra of gelatin and GelMA were collected with a Varian Mercury-400 NMR spectrometer (400 MHz). All spectra were measured at room temperature. D_2_O was used as a solvent, and the concentrations of both gelatin and GelMA were 10 mg/mL. The chemical shifts are presented in parts per million, downfield from the TMS standard. The proton signal of residual D_2_O was used as a reference.

### 2.4. Synthesis of Core-shell Nanogel (Amine-NG)

The core-shell nanogel (Amine-NG), with a pNIPAM core and a p(NIPAM-*co*-APMA) shell, was synthesized according to a previously reported approach with some modifications [33]. Briefly, Amine-NG was synthesized through a two-step precipitation polymerization. The reaction was conducted in 150 mL water and at a total monomer and cross-linker concentration of 140 mM; the molar composition is shown in Table 1. In a 250-mL three-necked round-bottom flask equipped with a magnetic stirrer, a reflux condenser, and a nitrogen in- and outlet, the monomer NIPAM, cross-linker BIS, surfactant CTAB, and fluorescent dye MRB were dissolved in 95 mL water. After degassing the mixture for 1 h with N_2_, the solution was heated up to 70 °C while stirring. The radical polymerization was initiated by injecting 5 mL of a degassed solution of the initiator AMPA V50 into the reaction mixture. The initiation of polymerization was indicated by the occurrence of turbidity. The reaction solution was stirred under a nitrogen atmosphere for 30 min at 70 °C. Meanwhile, a mixture of NIPAM, BIS, CTAB, MRB, and the comonomer APMA was dissolved in 50 mL water and degassed with N_2_ for 1 h. Subsequently, the comonomer mixture was slowly added to the reaction with a syringe (0.2 mL/min) to induce shell synthesis. The reaction proceeded for 6 h at 70 °C under a nitrogen atmosphere and stirring (300 rpm), and then was cooled down to room temperature under continuous stirring. The obtained nanogel was purified by dialysis (MWCO = 3500 Da) against water for one week; the water was changed twice per day. The purified nanogel was freeze-dried for further use.

### 2.5. Synthesis of Methacryloyl-Functionalized Nanogel (MA-NG)

To obtain the desired photo cross-linking capability, Amine-NG was functionalized with methacryloyl groups by modifying the primary amine groups with MA. As is similar to the synthesis method of GelMA, 0.2 g Amine-NG was dissolved in 10 mL water, and 0.4 g MA was slowly added. The pH was kept at 7 by adding a NaOH solution (0.5 M) during the reaction, which reacted for 6 h under constant stirring at room temperature. Afterward, the reaction mixture was diluted four times and then transferred to dialysis tubing of 3500 Da MWCO and dialyzed against ultrapure water for 7 days, to remove the excess methacrylic anhydride and methacrylic acid. The dialysis water was changed twice daily, followed by freeze-drying to obtain the purified functionalized nanogel (MA-NG).

### 2.6. Transmission Electron Microscopy (TEM) of Nanogels

The morphologies of the nanogels were observed under a Philips CM120 Microscope coupled to a 4k CCD camera using an acceleration voltage of 120 kV. All the samples were negatively stained with uranyl acetate and drop-casted on a carbon film-coated Cu grid.

### 2.7. Dynamic Light Scattering and Zeta Potential Measurements of Nanogels

The hydrodynamic diameters (D_h_) and polydispersities of the nanogels were determined by dynamic light scattering (DLS). The measurements were performed with a Malvern ZetaSizer Nano ZS ZEN3600 equipped with a temperature controller. The scattering detector was positioned at a fixed scattering angle of 173°. The concentrations of nanogel dispersions were around 0.1 mg/mL in water. Hydrodynamic diameters were calculated from the diffusion coefficients, using the Stokes−Einstein equation. The polydispersity index is established by the accumulated analysis method. Temperature-dependent measurements were performed at a range of 20–44 °C, with 2 °C intervals. Before the data collection of each temperature, the sample was allowed to equilibrate for 3 min at the proper temperature. Each data point is an average of three successive size measurements, which themselves consisted of 11–15 measurements.

Zeta potential measurements were performed with the same instrument at 20 °C. The concentrations of nanogel dispersions were around 0.01 mg/mL. The final Zeta potentials were a result of the average of three successive measurements. 

### 2.8. Potentiometric Titration of Nanogels

To determine the amount of incorporated amine comonomer within the Amine-NG, and the degree of functionalization of MA-NG, a potentiometric titration method was used to determine the number of primary amine groups [33]. The potentiometric titrations were conducted on a Metrohm 702 SM Titrino titrator at room temperature. A representative procedure can be described as follows: approximately 20 mg of Amine-NG or MA-NG was dispersed in 50 mL of water and transferred into the titration cell. The pH was adjusted to approximately 10 with 0.1 M NaOH. After an equilibration time of 15 min, the titration was performed by the addition of 0.1 M HCl in increments of 2 µL, and the pH was simultaneously measured with a Metrohm combined glass electrode. The amount of incorporated amine was calculated from the dependence of the pH on the volume of the added titrant. At least three independent experiments were performed for each sample tested.

### 2.9. Preparation of MA-NG-GelMA Composite Hydrogels and Scanning Electron Microscopy (SEM) Analyses

The LAP was used as a water-soluble UV photo-initiator to form the MA-NG-GelMA hydrogels by photopolymerization. In brief, 0.5% (*w/v*) of MA-NG was fully dispersed in water, and then 10% (*w/v*) of GelMA and 0.5% (*w/v*) of LAP were added at 30 °C until fully dissolved. The mixed solution was added into a cylindrical PDMS mold and covered with a glass slide, and was subsequently irradiated by UV light for 5 min at a wavelength of 365 nm using a Spectrolinker XL 1500 UV source (Spectronics Corp.) The UV lamp provided an intensity between 2300 µW/cm^2^ and 1100 µW/cm^2^. Pure GelMA hydrogels were prepared by the same procedure, mixing only 10% (*w/v*) of GelMA and 0.5% (*w/v*) of LAP in water as a control group.

To investigate the surface topography of MA-NG-GelMA hydrogels and GelMA hydrogels, the obtained hydrogels were lyophilized and broken manually to expose their cross-sections. Then, the broken samples were sputter-coated with ~20 nm gold, in order to improve conductivity. The microstructure was observed with a Helios G4 CX DualBeam in secondary electron (SE) mode, with a voltage of 5 kV and a current of 0.34 nA. 

### 2.10. Printability in 3D of MA-NG-GelMA and Confocal Laser Scanning Microscopy

To test the 3D printing capability, two inks (MA-NG-GelMA ink and pure GelMA ink) were prepared as described in Section 2.9. Both inks were then loaded into syringes topped with 1-inch Allevi’s 25G to 30G nozzles. The 3D printing was performed on a glass slide that had been pretreated with 3-(trimethoxysilyl)propyl methacrylate to promote bonding of GelMA, using a BioBots 1 3D bioprinter. Figure building is performed in 3D Builder, and 3D image slicing is achieved using the program Repetier Host. The exerted pressure was varied, depending on the observed material viscosity and temperature, to optimize the extrusion rate. Printed constructs were cross-linked by irradiating them with a UV lamp (405 nm wavelength, 7 mW/cm^2^) both during the printing and afterward for an additional 5 min.

To investigate the nanogel’s incorporation within the GelMA network, alternating vertical lines of MA-NG-GelMA and pure GelMA were printed. Since the nanogel was labeled with the fluorescent dye MRB, the confocal microscopy images of printed structures were obtained with a Leica TCS SP2 confocal laser scanning microscope.

### 2.11. Fluorescence Spectroscopy of MA-NG-GelMA and Amine-NG-GelMA Composite Hydrogels

**Scheme 1:**
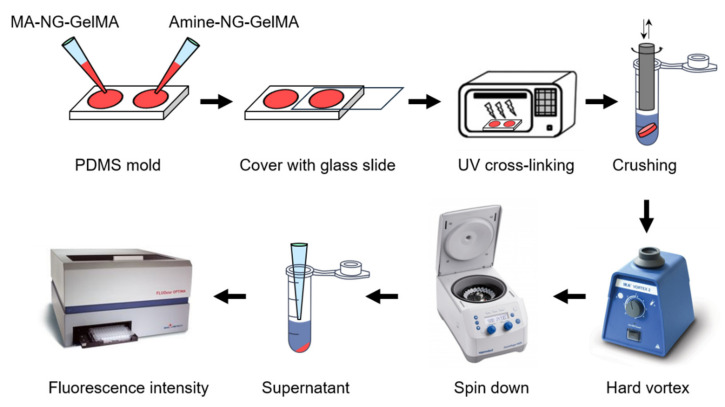
Schematic representation of the procedure used for the determination of covalent bonding of MA-NG inside the GelMA network. GelMA is dissolved in MA-NG or Amine-NG solution and is mixed with LAP, before casting it into a PDMS mold. The casting mold is then covered with glass slides and transferred into a UV cross-linker, to enable the formation of the hydrogels by photo-polymerization. The obtained hydrogels are crushed and vortexed vigorously in water, followed by centrifugation to spin down the hydrogel fragments. After taking out the supernatant, the fluorescence intensity of the supernatant is measured with a plate reader.

To prove the covalent bonding of MA-NG within the GelMA network, Amine-NG was incorporated into the GelMA hydrogel as a comparison, by the same method as the MA-NG-GelMA hydrogel preparation described in Section 2.9. As shown in Scheme 1, after UV cross-linking, both the Amine-NG-GelMA hydrogel and MA-NG-GelMA hydrogel were crushed and vortexed vigorously in water, followed by centrifugation to spin down the hydrogel fragments. Afterward, the fluorescence intensity of the supernatant was measured at 25 °C using a Synergy H1 Multi-Mode Reader at an excitation wavelength of 548 nm and an emission wavelength of 580 nm. Ultrapure water was measured as a reference.

## 3. Results and Discussion

### 3.1. Synthesis and Characterization of GelMA

GelMA was synthesized by binding the methacryloyl groups on the gelatin surface through a covalent coupling with the reactive primary amine groups of the lysine residues, as shown in Figure 1a. To obtain a high methacrylation degree, 0.6 g of methacrylic anhydride was added per gram of gelatin [34]. The degree of functionalization (DoF) of gelatin was determined using ^1^H-NMR spectrometry [35]. As shown in Figure 1b, compared with the spectrum of unmodified gelatin, the GelMA sample showed new signals corresponding with the methacryloyl groups. The signals at around 5.4 and 5.6 ppm chemical shifts were assigned to the acrylic protons (2H) of the grafted methacryloyl group (indicated by a black arrow), and the signal at 1.9 ppm was attributed to the methyl group (3H) of the grafted methacryloyl group (indicated by a red arrow). There was a decrease of intensity in the signal around 3.0 ppm, which was assigned to the lysine methylene (2H) of gelatin (indicated by blue arrows). The decrease of the integrated signal was used to calculate the DoF as the primary amine of lysine is the target site for the reaction, although minor reactions occurred with other reactive groups than amine groups in gelatin. The spectra were normalized by the aromatic moieties (5H) of phenylalanine signals, around 7.3 ppm, as an internal reference since they were not modified by MA during the reaction. The estimated DoF is 75%, which is consistent with the previously reported number [34].

### 3.2. Synthesis and Characterization of Nanogels

**Figure 2:**
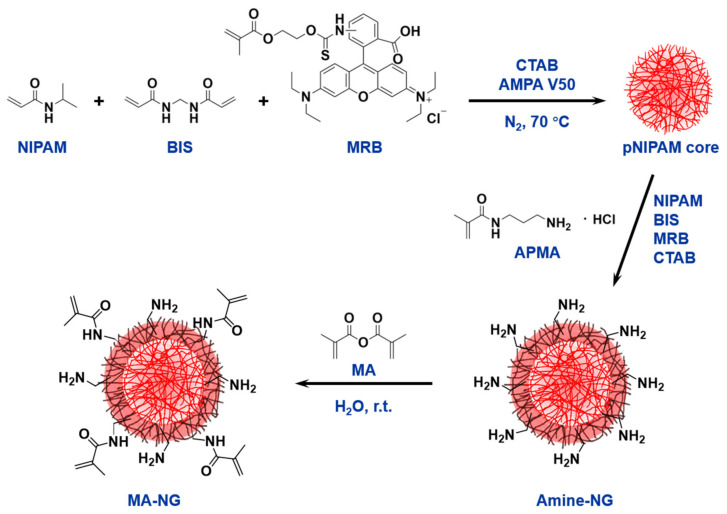
Synthesis route of Amine-NG and MA-NG.

To be able to covalently incorporate the nanogel inside the GelMA hydrogel matrix, a methacryloyl-functionalized nanogel (MA-NG) was prepared by coupling MA with primary amine moieties on the surface of the nanogel, to obtain the cross-linking capabilities. Therefore, a primary amine-functionalized core-shell nanogel (Amine-NG) containing a pNIPAM core and a p(NIPAM-*co*-APMA) shell was firstly synthesized via two-step precipitation polymerization, using the cationic initiator AMPA V50 and the cationic surfactant CTAB to stabilize the particles, as shown in Figure 2. The fluorescent dye MRB was introduced, to label the nanogel for further characterization.

**Figure 3:**
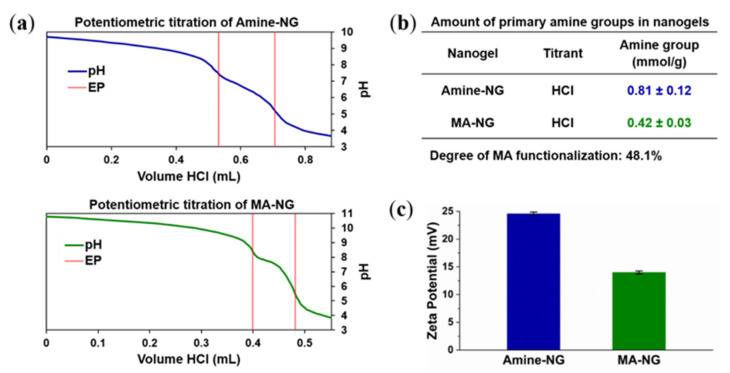
(**a**) Potentiometric titration of Amine-NG and MA-NG at 25 °C; (**b**) the number of primary amine groups in nanogels determined by potentiometric titration; (**c**) the Zeta potential of Amine-NG and MA-NG in water at 25 °C.

The absolute amount of incorporated amine groups of Amine-NG and MA-NG was determined by potentiometric titration. The representative titration curves are shown in Figure 3a; the potentiometric titration of both Amine-NG (blue line) and MA-NG (green line) exhibit two regions. The first region, down to V_HCl_ = 0.531 mL of Amine-NG and V_HCl_ = 0.399 mL of MA-NG, corresponds to the titration of any excess NaOH that is present, whereas the second region, until V_HCl_ = 0.706 mL of Amine-NG and V_HCl_ = 0.481 mL of MA-NG, can be assigned to the protonation of the amine groups. The number of primary amine groups can be recalculated from the added volume of the titrant HCl between these two equivalence points (EP). The amount of amine was determined to be 0.81 ± 0.12 mmol/g for Amine-NG, and 0.42 ± 0.03 mmol/g for MA-NG, as shown in Figure 3b. Accordingly, the degree of MA functionalization is around 48.1%, calculated from the amount of amine in Amine-NG and MA-NG. To further prove the functionalization with MA, the Zeta potentials of both Amine-NG and MA-NG were measured; the results are shown in Figure 3c. The Zeta potential of Amine-NG is around +25 mV, due to the used cationic initiator and the incorporation of primary amine groups on the surface of the nanogel. After MA functionalization, the Zeta potential of MA-NG decreased to +14 mV, which indicated a lower amount of primary amine groups present, and successful modification with methacryloyl groups.

**Figure 4:**
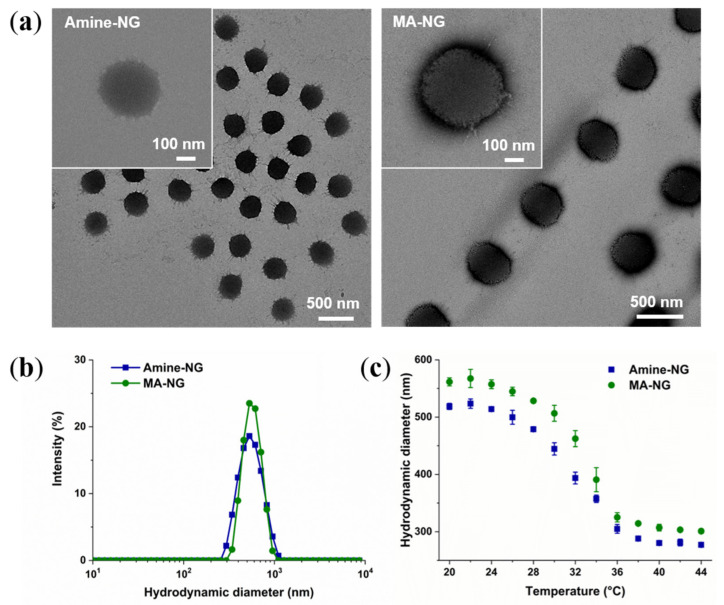
(**a**) TEM images of Amine-NG and MA-NG; (**b**) size distribution by the intensity of Amine-NG and MA-NG in aqueous media, determined by DLS analysis in water at 20 °C; (**c**) hydrodynamic diameter as a function of the temperatures of Amine-NG and MA-NG, obtained from DLS measurements.

The size and morphology, hydrodynamic properties, and behavior in aqueous media of the nanogels were investigated by TEM and DLS analysis. The representative TEM images, hydrodynamic size distribution, and the temperature-dependent hydrodynamic diameter curves of Amine-NG and MA-NG, are shown in Figure 4. Both Amine-NG and MA-NG were spherical in shape with fuzzy edges, monodispersed. The diameter in the dry state was similar for both nanogels: 354 ± 20 nm for the Amine-NG, and 415 ± 14 nm for MA-NG, as shown in Figure 4a. The slight difference between the sizes may come from the artifacts during the negative staining or drying process. The hydrodynamic diameters of both nanogels in water displayed a narrow size distribution, as shown in Figure 4b; the average hydrodynamic diameter is 519 ± 5 nm for the Amine-NG and 561 ± 7 nm of MA-NG. Figure 4c shows the variation of the nanogels in hydrodynamic diameter according to temperature since the pNIPAM segment is temperature-responsive. The volume-phase transition temperature (VPTT) is around 34 °C for Amine-NG and 33 °C for MA-NG. The slightly higher VPTT of both nanogels compared to pure pNIPAM nanogel (around 32 °C) is due to the presence of the amine functional groups, which are more hydrophilic and display charge repulsion in the protonated form, thus increasing the VPTT. However, after MA functionalization, the VPTT slightly decreases to 33 °C, due to the consumption of amine groups and the introduction of more hydrophobic methacryloyl groups (compared to amine groups). It is notable that the hydrodynamic diameter of nanogels is slightly increased after MA functionalization over the whole temperature range, which might be attributed to the contribution of steric hindrance from the methacryloyl groups.

### 3.3. Preparation and Morphology of MA-NG-GelMA Composite Hydrogel

**Figure 5:**
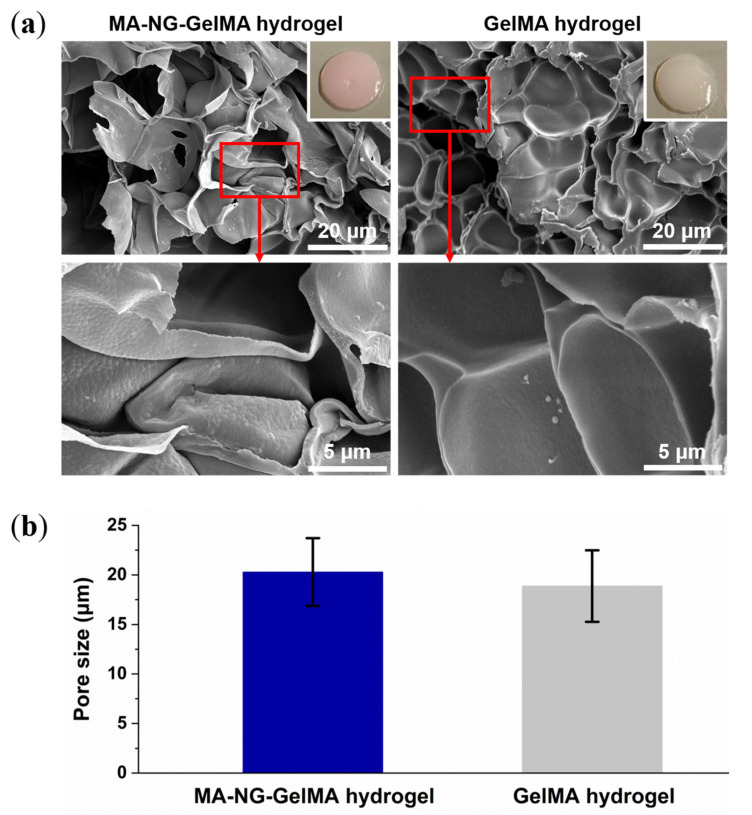
(**a**) SEM images of MA-NG-GelMA hydrogel and pure GelMA hydrogel. Photographs of MA-NG-GelMA hydrogel (pink) and pure GelMA hydrogel (transparent) were inset. (**b**) Pore sizes of the MA-NG-GelMA hydrogel and pure GelMA hydrogel.

The MA-NG-GelMA composite hydrogel (0.5% MA-NG + 10% GelMA) and pure GelMA hydrogel (10% GelMA) were prepared in a cylindrical PDMS mold, LAP (0.5%); a recently developed alternative water-soluble photo-initiator [36] was used to initiate the photopolymerizations under UV light. As shown in the inserted photograph in Figure 5a, after UV cross-linking, the color of the GelMA hydrogel is transparent, whereas the MA-NG-GelMA composite hydrogel is pink, which comes from the introduced fluorescent dye MRB in the nanogel.

To investigate the morphology of the porous structure within the hydrogel, the prepared MA-NG-GelMA composite hydrogel and pure GelMA hydrogel were lyophilized, and the images of the cross-sectional microstructures were observed using SEM. As shown in Figure 5a, both the GelMA hydrogel and MA-NG-GelMA composite hydrogel presented a highly porous structure, with irregular pore shape and different pore sizes, due to the syneresis phenomenon during the lyophilization process. The average pore size of both hydrogels was counted using the ImageJ software, the results of which are shown in Figure 5b. The average pore sizes were inversely related to the degree of methacryloyl substitution and the concentration of the GelMA solution [7]. In this study, the DoF of the used GelMA was 75%, and the concentration of GelMA solution was 10%. The pore diameters were 20.3 ± 3.4 μm of the MA-NG-GelMA composite hydrogel, and 18.9 ± 3.6 μm of the pure GelMA hydrogel, which is comparable with the previously reported value with a similar DoF (23.6 ± 5.85 μm with 73.2% DoF of pure GelMA hydrogel) [37].

To evaluate the introduction and distribution of MA-NG nanogels inside the GelMA network, high-magnification SEM images were acquired, as shown in Figure 5a (second row). It is worth noting that in the contrary of the smooth surface of the pore wall of the pure GelMA hydrogel, the MA-NG-GelMA composite hydrogel showed a rough surface of the pore wall due to the nanogel incorporation. It can be observed clearly, from the higher magnification insets, that a large amount of MA-NG was wrapped within the GelMA network, covering the surface of the pore wall of the composite hydrogel. However, not all the nanogels were evenly distributed over the whole surface, but were instead in a sporadically clustered form, which is possibly because of insufficient mixing of the nanogel solution and GelMA.

### 3.4. Covalent Bonding of MA-NG within MA-NG-GelMA Hydrogels

**Figure 6:**
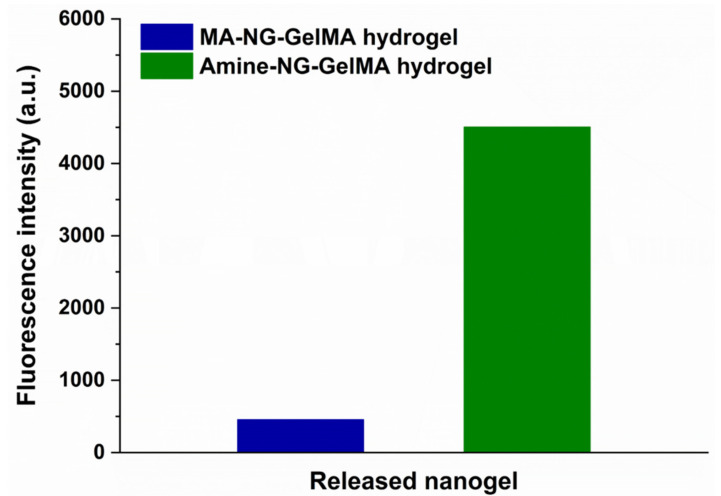
Fluorescence intensity of qualitative release of nanogels from MA-NG-GelMA hydrogel and Amine-NG-GelMA hydrogel in the supernatant of centrifuged hydrogel fragment, after crushing and vortexing vigorously in water.

An important key point of nanoparticle-composite GelMA hydrogels for drug delivery and other biomedical applications is the need for stability of the hierarchical system. Therefore, in this study, the nanogels were covalently bound to the GelMA network by photopolymerization. To prove the covalent bonding, a release experiment of nanogel from the GelMA hydrogel was conducted, as shown in Scheme 1. The Amine-NG was incorporated in the GelMA hydrogel (Amine-NG-GelMA hydrogel) in the same way as the MA-NG-GelMA hydrogel, as a control group. For normalization of the fluorescence intensity of both nanogels, the fluorescence spectra of Amine-NG and MA-NG were obtained using a plate reader (Figure S1 in the Supplementary Material). The fluorescence intensity of the released nanogels in the supernatant was measured by plate reader; the normalized results are shown in Figure 6. It can be seen that the Amine-NG was dramatically released from the hydrogel, compared to the MA-NG (around 10 times), after the crushing and vigorous vortex treatment. This difference indicates that the MA-NG was covalently bound to the GelMA network, due to the photopolymerization capability induced by methacryloyl functional groups, whereas the Amine-NG was only blended into the GelMA network under the same conditions.

### 3.5. Printability in 3D of MA-NG-GelMA

**Figure 7:**
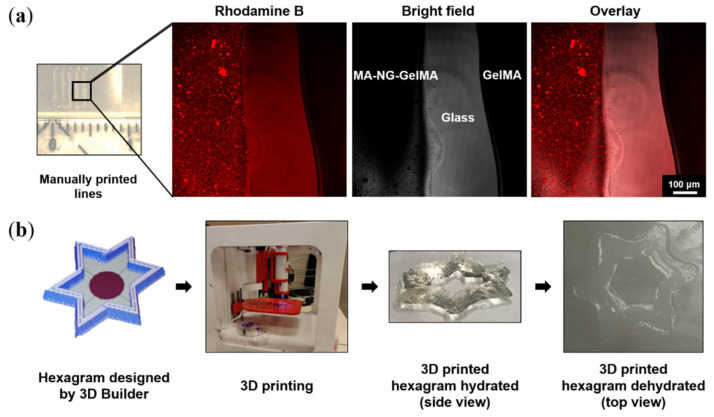
(**a**) 3D-printed alternating vertical lines and the representative confocal images of MA-NG-GelMA and pure GelMA; (**b**) 3D printing process of MA-NG-GelMA in a hexagram shape.

The main focus of this research was to develop a 3D-printable nanogel-GelMA composite hydrogel to achieve a hierarchical platform for the potential applications of regenerative medicine and tissue engineering. The 3D printing procedure was performed at room temperature, and the printed hydrogel was cross-linked by irradiating it with UV light (405 nm wavelength, 7 mW/cm^2^) during the printing, and afterward for an additional 5 min. Before printing with predesigned patterns, alternating vertical lines of pure GelMA and MA-NG-GelMA were printed for the 3D printing tests by loading two inks into two syringes separately and manually controlling the extrusion process. In order to observe the morphology of the printed constructs, the printed adjacent lines were imaged using a confocal microscope, as shown in Figure 7a. The printed MA-NG-GelMA hydrogel line (left) showed a pronounced fluorescent signal contributed from the MRB-modified nanogel, while the printed GelMA hydrogel line (right) had no fluorescent signal at all. However, the fluorescent signal distribution of the MA-NG-GelMA hydrogel line was not uniform, showing certain agglomeration patterns, which is consistent with the sporadically clustered form of the nanogel inside the GelMA network observed by the SEM analysis.

To further test the printability of the MA-NG-GelMA ink, a 3D pattern with a hexagram shape was designed with 3D Builder and printed with MA-NG-GelMA ink; the printing process and printed construct are shown in Figure 7b. The printed hydrogel presented homogeneity and stability after UV cross-linking, and maintained the hexagram shape after dehydration for a few minutes in air. These results further confirmed that the prepared MA-NG-GelMA ink is 3D-printable, and that these kinds of hybrid approaches of ink formation are an upcoming new trend to create more functional hydrogel systems [38].

## 4. Conclusions

In this study, we introduced a simple, versatile method to manufacture hierarchical nanogel–GelMA composite hydrogels. To achieve the covalent bonding of the nanogel within the GelMA network, the nanogel was modified with methacryloyl groups on the surface to obtain the necessary photo-cross-linking ability by coupling MA with the primary amine groups on the shell of the nanogel. The nanogel–GelMA composite hydrogel was formed by mixing GelMA and LAP with the nanogel solution, followed by UV irradiation. The SEM images clearly showed that the nanogel was successfully embedded inside the GelMA network. To investigate the covalent bonding and the stability of MA-NG inside the GelMA hydrogel, a release experiment was conducted to compare it with noncovalent introduced Amine-NG in GelMA hydrogel. The results showed a significant release of noncovalent Amine-NG from the GelMA hydrogel, whereas the covalently bound MA-NG showed only a slight release. Finally, the 3D-printability was tested with a BioBot 1 3D printer, and the printed structure was visualized under confocal microscopy, which showed inhomogeneous distribution with certain aggregation patterns, due to the insufficient mixing of the nanogel and GelMA polymer. Overall, we have provided a new concept in the 3D printing of a stable hierarchical hydrogel system, by introducing nanocarrier nanogel in the micropore GelMA hydrogel network. The developed system has great potential to achieve the in situ delivery and controllable release of bioactive molecules to encapsulated cells, which could lead to the direct engineering of a mature controllable 3D cell culture system in vitro that is close to the native tissue analog and is thus more suitable for transplantation. 

## Figures and Tables

**Table 1:** Molar composition of the core-shell nanogel (Amine-NG) reaction mixture, and the amounts of the chemicals used in the synthesis.

Component	Chemical	Abbreviation	Mass (mg)	Molar Amount (mmol)	Molar Content (%)
Core	Monomer	NIPAM	1505	13.3	95
	Cross-linker	BIS	108	0.7	5
	Surfactant	CTAB	4	0.011	-
	Dye	MRB	10	0.015	-
	Initiator	AMPA V50	54	0.2	-
Shell	Monomer	NIPAM	673	5.95	85
	Comonomer	APMA	125	0.7	10
	Cross-linker	BIS	54	0.35	5
	Surfactant	CTAB	2	0.005	-
	Dye	MRB	5	0.008	-

## Data Availability

The data presented in this study are available on request from the corresponding author.

## Supplementary Materials

The following are available online at https://www.mdpi.com/article/10.3390/polym13152508/s1, Figure S1: Fluorescence spectra of Amine-NG and MA-NG.
