# Supplementary material for: 3D-Printable Hierarchical Nanogel-GelMA Composite Hydrogel System
Source: Polymers (Basel). 2021 Jul 29;13(15):2508. doi: 10.3390/polym13152508 (PMC8348806; doi:10.3390/polym13152508)
Supplement: Supplementary file 1 [file polymers-13-02508-s001.zip › polymers-1312863-supplementary.pdf]

polymers

MDPI

Article

3D-Printable Hierarchical Nanogel-GelMA Composite Hydrogel System

Guangyue Zu [1], Marnix Meijer [1], Olga Mergel [1], Heng Zhang [2] and Patrick van Rijn [1,2] *

[1] Department of Biomedical Engineering, W. J. Kolff Institute for Biomedical Engineering and Materials Science, University of Groningen and University Medical Center Groningen, A. Deusinglaan 1, 9713 AV, Groningen, The Netherlands
[2] Zernike Institute for Advanced Materials, University of Groningen, Nijenborgh 4, 9747 AG Groningen, The Netherlands
* Correspondence: p.van.rijn@umcg.nl

Supplementary Materials

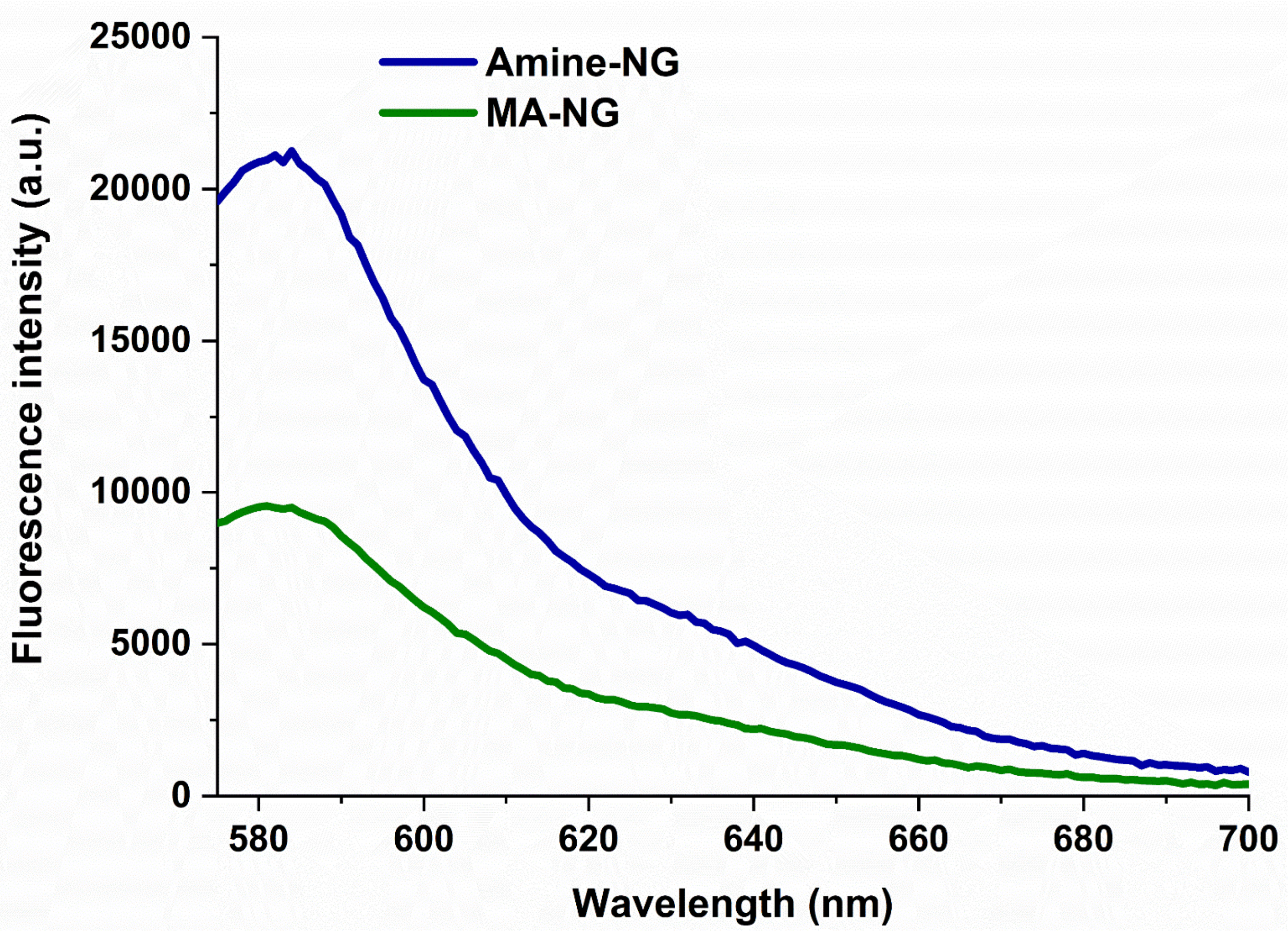

Figure S1. Fluorescence spectra of Amine-NG and MA-NG in water.
